# Prioritization of therapy uncertainties in Dystrophic Epidermolysis Bullosa: where should research direct to? an example of priority setting partnership in very rare disorders

**DOI:** 10.1186/1750-1172-8-61

**Published:** 2013-04-22

**Authors:** Paula Davila-Seijo, Angela Hernández-Martín, Evanina Morcillo-Makow, Raúl de Lucas, Esther Domínguez, Natividad Romero, Eva Monrós, Marta Feito, Luis Carretero, Bea Aranegui, Ignacio García-Doval

**Affiliations:** 1 Department of Dermatology, Complexo Hospitalario de Pontevedra (CHOP), Doutor Loureiro Crespo 2, Pontevedra, 36001, Spain; 2 Department of Pediatric Dermatoloy, Hospital Infantil Niño Jesús, Madrid, Spain; 3 The Dystrophic Epidermolysis Bullosa Research Association (DEBRA), Spain, Spain; 4 Pediatric Dermatology section, Department of Dermatology, Hospital Universitario de La Paz, Madrid, Spain; 5 DEBRA member, Madrid, Spain; 6 Department of Dermatology, Clínica Universitaria de Navarra, Madrid, Spain; 7 Research Unit, Fundación AEDV, Academia Española de Dermatología y Venereología, Ferraz 100, 1° izda, Madrid, 28008, Spain

**Keywords:** Dystrophic Epidermolysis Bullosa, Treatment uncertainties, Therapy uncertainties, Priority Setting Partnership, Patient participation, Research prioritization

## Abstract

**Background:**

Dystrophic Epidermolysis Bullosa (DEB) is a rare genodermatosis (7 cases per million) that causes blisters and erosions with minor trauma in skin and mucosa, and other systemic complications. A recently updated systematic review showed that the research evidence about DEB therapies is poor. As new trials in DEB are difficult and expensive, it is important to prioritizise research that patients and clinicians consider more relevant.

**Objectives:**

To describe and prioritize the most important uncertainties about DEB treatment shared by patients, carers and health care professionals (HCPs) in order to promote research in those areas.

**Methods:**

A DEB Priority Setting Partnership (PSP) was established, including patients, carers and HCPs. DBE uncertainties were gathered from patients and clinicians, and prioritized in a transparent process, using the methodology advocated by the James Lind Alliance.

**Results:**

In the consultation stage, 323 uncertainties were submitted by 58 participants. Once the duplicated and non-treatment uncertainties were removed, the remainder were reduced to a list of 24 most voted questions. These 24 uncertainties were prioritized in a final workshop where a balanced number of patients, carers and HCPs selected the top 10 therapy uncertainties. The final list includes interventions in wound care, itch and pain management, treatment and prevention of syndactyly, cancer prevention and future promising therapies.

**Conclusions:**

The final list of the top 10 treatment uncertainties on the management of DEB provides guidance for researchers and funding bodies, to ensure that future research answers questions that are important to both clinicians and patients. The method proposed by the James Lind Alliance is feasible for very rare disorders.

## Background

Dystrophic Epidermolysis Bullosa (DEB) is a rare genetic skin disease (7:1,000,000 live births) [1] that produces extreme fragility of skin and mucosa and blistering after friction [2-4], as well as other systemic complications (such as malnutrition, growth delay, infection, heart and renal disease or skin-cancer) [5-12]. The patients have to live with constant pain, scarring sequelae and eventually disfigurement, disability and often, early death [13-16].

A recently updated systematic review of therapies for inherited forms of Epidermolysis Bullosa (EB) identified, up-to December 2011, seven randomized clinical trials (RCTs) evaluating seven different interventions for decreasing number of skin lesions [17,18]. None of the studied medications showed a significant effect. Most of the studies were poorly reported, and of small size, highlighting the difficulties of recruiting patients for trials. Given this lack of experimental evidence, many clinical questions have an uncertain answer.

As DEB is a rare disease, multi-centre trials with international collaboration are likely to be needed to solve therapeutic uncertainties. As the research budget is limited and the number of uncertainties (unanswered clinical questions) is large, prioritizing the most important ones requiring research in DEB is essential [19]. The traditional way of setting the research agenda is usually led by researchers, academia and pharmaceutical industry, but these groups might not focus on the same areas as patients, carers and health care professionals (HCPs) [20]. This mismatch has been the focus of recent attention [21], and the James Lind Alliance, funded by the UK National Institute for Health Research [22], has developed a method (Priority Setting Partnerships (PSP) [23] to identify and prioritize for research the treatment uncertainties which patients, carers and clinicians agree are the most important. The method consists of several steps, including joining representative participants, collecting uncertainties from them, excluding those that can be answered with available research, and ranking the remaining ones, the real uncertainties. There are few examples of the use of this method in dermatology [24,25], and to our knowledge this has not been applied to very rare disorders, where prioritization might be especially important.

## Materials and methods

The aim of the DEB PSP was to identify treatment uncertainties that are relevant for patients and clinicians and to prioritize them in a transparent process that can help steer future clinical research.

**Figure 1:**
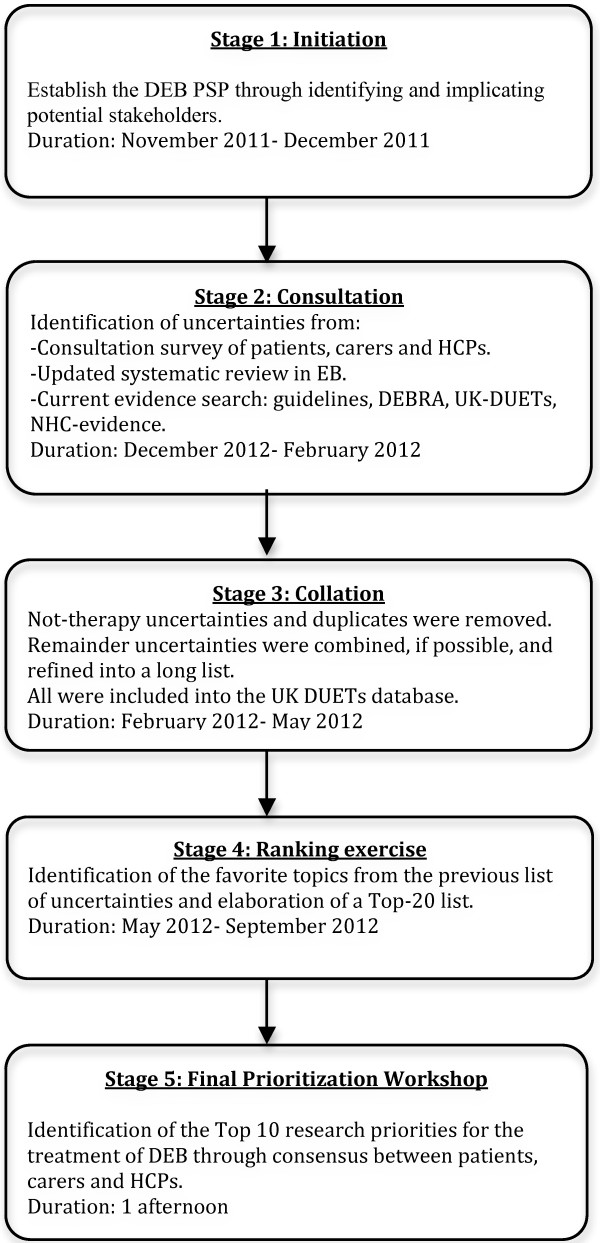
**Summary of the methodology used in the PSP.** DEB PSP, Dystrophic Epidermolysis Bullosa; HCPs, heath care professionals; EB, Epidermolysis Bullosa; DEBRA, the Dystrophic Epidermolysis Bullosa Research Association; UK-DUETs, (UK Database of Uncertainties about the Effects of Treatment).

The five stages of the PSP (see Figure 1 for a summary of the methodology used), took place between November 2011 and October 2012.

The DEB PSP adopted the methods promoted by the James Lind Alliance adapted to meet the needs of this PSP.

The Steering Group comprised eight people with experience in DEB including patients/carers, a representative from the Dystrophic Epidermolysis Bullosa Research Association Spain (DEBRA Spain), health care professionals (dermatologists and nurses) and researchers/epidemiologist.

### Stage 1: Initiation

The aim of this stage was to establish the DEB PSP through identifying and implicating potential stakeholders. We contacted potential partner organizations and individuals using the Steering Group members’ networks and through the DEBRA Spain´s register of affiliates. We tried to ensure that all the following groups were represented: patients affected with DEB, carers of children with DEB, physicians involved in EB care, nurses, psychologist and other professionals related with treatment and care of people with DEB.

Organizations approached at this stage were: The Dystrophic Epidermolysis Bullosa Research Association of Spain (DEBRA Spain) and the Spanish Academy of Dermatology and Venereology (AEDV).

To identify experts on the disease we used information from DEBRA, DEBRA associates, physicians working in centers with expertise in DEB care reference, and contacted all authors of papers included in Medline, with an address in Spain, and reporting a minimum of three patients. We also asked all experts to identify other experts and justify their selection. To identify patients and carers we included all members of DEBRA Spain with DEB (as many of them were children, their parents were contacted).

### Stage 2: Consultation survey: collection of treatment uncertainties

The aim of this stage was to collect treatment uncertainties using the following question: “What question(s) about DEB treatment would you like to be answered by research?” An pre-piloted online survey using SurveyMonkey was carried out to collect uncertainties from patients, carers and healthcare professionals [26]. The survey and informative documents were revised by patients in order to guarantee readability and easy understanding. A paper copy sent after a phone call was used with to some participants who had no access to an e-mail account. Details of the project and an informed consent were also provided as attached documents.

Each participant submitted a maximum of twenty treatment uncertainties. We additionally obtained information about the category of each participant (patient, carer, physician, nurse or other).

Additional treatment uncertainties were identified from existing evidence: the “Updated systematic review of randomized controlled trials of treatments for inherited forms of Epidermolysis Bullosa” [17,18], NHS Evidence [27], DEBRA America web page (Section Understanding EB) [28] and other country-based or consensus guidelines [4,29-31].

As a step required by the JLA method, we also contacted UK-DUETs (UK Database of Uncertainties about the Effects of Treatments) to include previously registered uncertainties in DBE [32].

### Stage 3: Collation of treatment uncertainties

The aim of this stage was to review the treatment uncertainties that we collected in the previous stage. Firstly, we removed any uncertainty not related with therapy, comments or personal queries. Then, we tried to refine the uncertainties into a standard PICO format (Patients, Intervention, Comparison, Outcome). Finally we combined any similar uncertainties. For example the uncertainty “What is the best treatment to control itch in DEB patients?” includes different systemic and topical drugs.

This stage was carried out by two researchers. The full list of refined uncertainties was checked by all members of the Steering Group to ensure that the meaning of the questions had not been modified, both during collation and after translation into English, for the next step.

Thereafter, the complete list of uncertainties was sent to UK-DUETs, reviewed and accepted for inclusion in the UK-DUETs database.

### Stage 4: Ranking exercise

The aim of this stage was to rank the uncertainties collected and collated in the previous stages in order to create a “Top-Twenty uncertainties” that were the most important for patients, carers and HCPs. We sent a link to a new on-line survey (or a paper copy) to all the participants, asking them to select a maximum of five main uncertainties. The order of the uncertainties in the survey was randomized in order to avoid response bias.

The results of this ranking were analyzed and sorted on a rank order by frequency of votes. An analysis by participants was also made to ensure that patients, carers and HCPs were equally represented in the final uncertainties´ compilation.

### Stage 5: Final prioritization workshop

The aim of this final stage was to identify the top 10 most relevant uncertainties for DEB, obtained by consensus between patients, carers and HCPs through a workshop using the nominal group technique advocated by the JLA. The participants were selected from individuals that had carried out previous stages of the DEB, from partner organizations and the Steering Group. Care was taken to ensure a balanced number of patients, carers and HCPs.

The workshop was half-day event, held at the Spanish Academy of Dermatology and Venereology offices in Madrid on 19 October 2012.

Participants were divided into three discussion groups, with five participants each, an even representation of the different groups (patients, carers and HCPs) and supervised by one facilitator who did not participate in the discussion but ensured that all individuals took part equally into the debate. All participants signed a declaration of absence of conflicts of interests.

Further details of the methods used during the DEB PSP are listed in the James Lind Alliance guidebook (http://www.jlaguidebook.org).

Given the rarity of the disease and some circumstantial problems, two of the participants in the workshop had to participate remotely, using videoconference (with Skype®) and with the support of a moderator.

## Ethics

This project was approved by the “Comité Ético de Investigacion Clínica”, Consellería de Sanidade, Xunta de Galicia, Spain (reference 2011/378).

### Statistical methods

Data from all stages were stored in Microsoft Excel 2008 and analyzed with Stata 12.1 software (StataCorp College Station, TX, USA). The survey was elaborated using the Survey Monkey on-line application [26].

## Results

### Stage 1: Initiation

**Figure 2:**
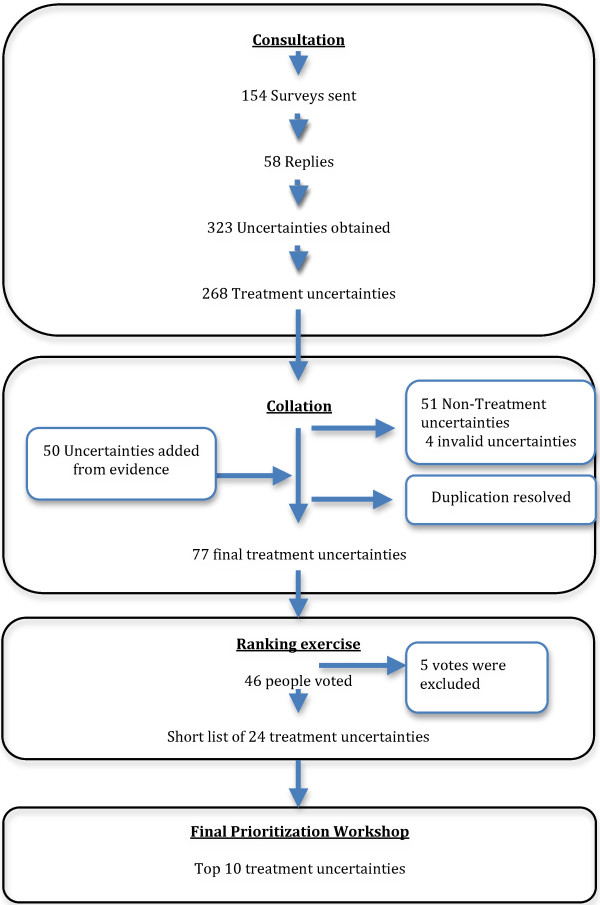
Summary of the results of the DEB PSP.

As organizations, we contacted DEBRA and the AEDV. The group of professionals included 29 participants, including dermatologists, surgeons, dentists, pediatricians, nurses, psychologists and a social worker. We contacted 125 patients- carers (Figure 2).

### Stages 2 and 3: Consultation and collation

Of the 154 surveys sent, 62 (40%) were returned. The response rate for patients and carers was 33% (41/125; 23 patients and 18 carers). 59% of HCPs consulted responded (17/29). 40% of responses (23/58) were from patients, 31% (18/58) were from careers and the remaining 29% (17/58) were from HCPs.

323 questions about DEB were obtained. The non-treatment questions (n=51) and invalid ones (n=4) were excluded. Once the duplicates were removed we obtained seventy-three (n=73) uncertainties. Additional uncertainties (n=50) extracted from the literature were added. The resulting 123 uncertainties were refined into a list of 77 uncertainties, all accepted in UK-DUETS [27], which were used in the next stage (Figure 2).

### Stage 4: Ranking exercise

A new survey was sent to the same individuals as in consultation stage (n=154). Forty-six people answered the survey (30%). The number of votes for each uncertainty ranged from 17 to 0.

28% (13/46) of the responders were patients, 33% (15/46) were carers and 39% (18/46) were HCPs.

Patients and carers were also asked to report a subjective degree of their disease severity in order to assure that patients of different severities were represented. 11% (3/11) described themselves as suffering from mild disease, 39% (11/28) had moderate disease, 39% (11/28) had severe DED and 11% (3/28) had very severe disease.

The range of age between the patients was 21 to 54 (median age 35.8 years) and the age range of affected represented by carers was 3 to 23 (median age 10.3 years).

At the end of this stage a list of 24 uncertainties was obtained (instead of 20, due to the presence of ties).

### Stage 5: Final prioritization workshop

The final workshop was attended by three patients (of different ages and gender), two carers, six HCPs and a representative of DEBRA Spain as well as three facilitators (see acknowledgements).

**Figure 3:**
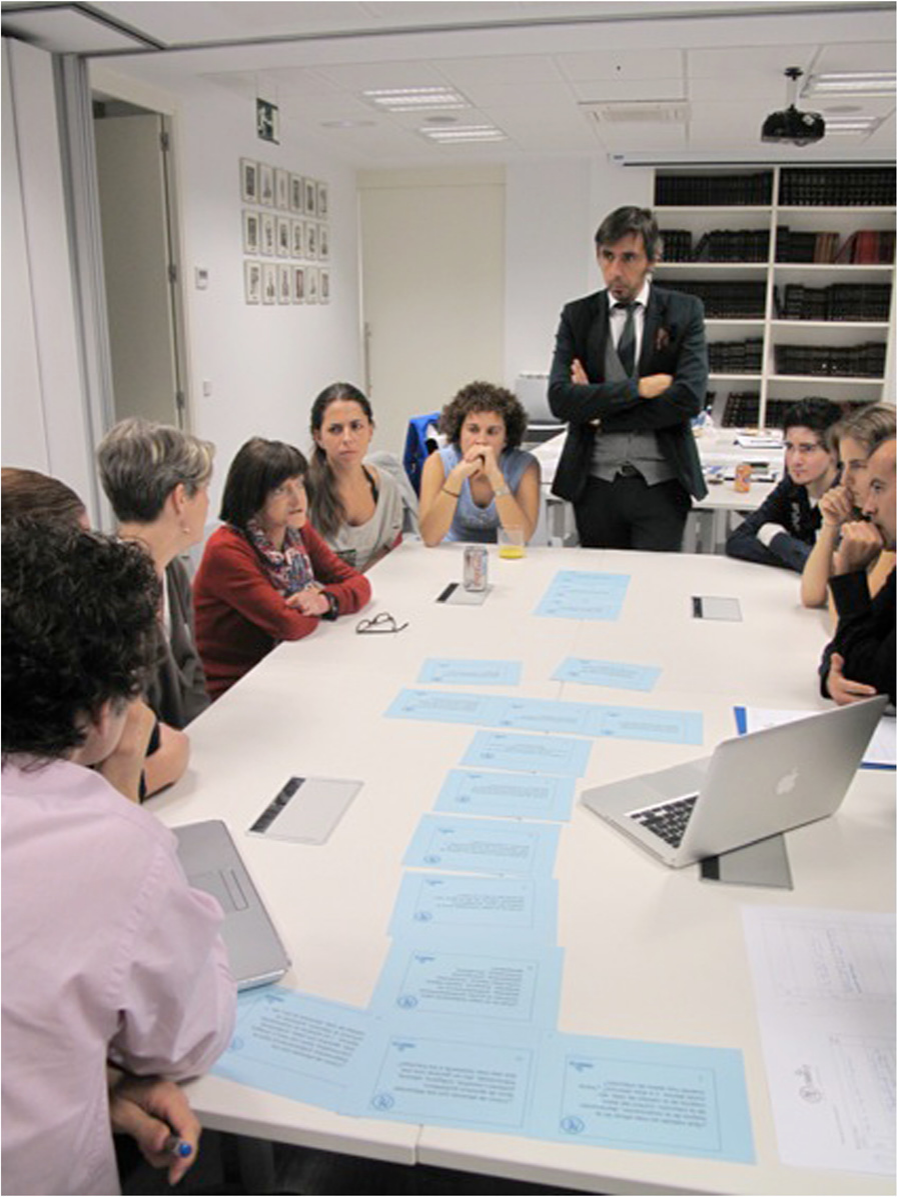
**Final prioritization workshop process.** All the participants, with the two remote participants using videoconference, during the final discussion. On the table, paper cards with each one of the top 10 uncertainties ranked from the most important to the least.

Each group independently selected their top ten uncertainties. Then the three lists were joined into an overall ranking that was examined by all participants. After a new discussion process, a “top ten” list was established by consensus (Figure 3). There was a high level of agreement amongst participants for the initial proposals in the three groups. 15 uncertainties (out of 24) got all the votes in the first round. The final top 10 ordered DEB therapy uncertainties are listed below:

1. Which wound care method obtains better outcomes (improved healing, decrease pain, improve quality of life, decrease smell, prevent infection) in patients with EB? Interventions include types of dressings (polyethylene, polyester plus petrolatum, hydrocolloid, collagen, hydrofiber, hydrogel, silicone…), topical antibacterial treatment (clorhexidine, bleach bath, vinegar bath, honey, antibiotics, silver dressings) and frequency of cure (daily or alternate days)?

2. What is the best treatment to control itch in DEB patients (sedating antihistaminics, non-sedating antihistaminics, topical menthol, topical corticosteroids, moisturizers, doxepin, gabapentine, cyclosporine, dronabinol, ondansetron)?

3. What is the best pain control strategy (analgesics, sedative drugs, addition of NaCl into the water) to decrease pain during wound care and bath in DEB patients?

4. How much does management in reference centers help patients with DEB (in terms of quality of life, avoiding complications and disability, cost-effectiveness)?

5. How effective is a "tumor early diagnosis protocol" in patients with DEB to decrease mortality, amputations and disability?

6. What are the long-term results of syndactyly surgery? Which is the best technique? How often should it be performed?

7. Which is the most effective method in avoiding or delaying syndactyly in patients with DEB? Including different types of bandages, dressings, gloves and splints, physiotherapy and occupational therapy.

8. What role might tissue engineering have in treating wounds in patients with DEB?

9. What role might stem cell therapy and bone marrow transplantation play in treating DEB?

10. What role might growth hormone play in decreasing growth delay and puberty delayed in DEB patients?

Some uncertainties were merged into one final uncertainty when there was a complete agreement between the participants.

## Discussion

Working with DEB patients and HCP during the PSP has highlighted the large number of uncertainties about the treatment that both groups have and share.

The final workshop showed what were the main research needs shared by patients, carers and HCP. These were, in decreasing order of importance:

1. Wound care. This was a fundamental issue for patients, carers and HCPs and is the one with more unanswered questions about it.

2. Itch. It is an important issue for all participants, leading to important discomfort and decreased quality of life.

3. Pain. Pain is a big problem for DEB patients. It was felt by participants that part of it could be solved by proper application of current knowledge, and involvement of pain management specialists in DEB care. However, it was also felt that there are some questions about pain therapy that were specific for the disease, such as treatment of pain during baths or during cures, that might require specific research. (For example, some authors report decreased pain after adding salt to the bath water).

4. Neoplasms, especially issues of early detection and treating squamous cell carcinoma.

5. Syndactyly. There is a lack of knowledge about what the best method in preventing or delaying syndactyly is as well as in correcting syndactyly once it is established.

6. New therapies. There was a shared thought about the importance of investigating new therapies in order to discover a cure for the disease and also find new techniques such as genetic engineering that could offer betters outcomes in the most common signs and symptoms of DEB. However these were rated lower than research on everyday problems.

It is also important to mention the perceived need to demonstrate the usefulness of reference centers in decreasing complications and increasing quality of life in order to encourage health authorities to develop these centers.

We are aware of that some of the questions prioritized may be broad, representing areas of concern more than specific questions. This will be less useful for planning research on them but, because there are so many uncertainties in each field, all participants agreed that they preferred a final list of uncertainties that reflects their most relevant concerns even at the expense of being less specific. However we believe that these areas of concern can be easily transformed into more specific ones for research. For example, the question about the best therapy to relieve itch in DEB patients could lead to many different questions that should also be prioritized: such as “do DEB patients who take doxepin experience less itch than those who do not?” or “is gabapentine more effective than dronabinol in treating the itch among DEB patients? or maybe “do DEB patients who use topical corticosteroids experiment less itch than those do not?

To date, twenty PSP have been conducted by the James Lind Alliance, mainly about common disorders [23-25]. To the best of our knowledge, the DEB PSP is the first PSP conducted about a very rare disease. In this kind of diseases, due to its infrequency and scarce investment in research it is particularly important to prioritize uncertainties. The main difference between this PSP and the previous conducted by the JLA may be resumed in several points:

Firstly, the lower number of patients and stakeholders: These make it more difficult to identify them, but they are easier to contact.

There was also difficulty in defining and finding expert clinicians. Unlike in common disorders, where most doctors will be experienced, and it is easy to find experts, for rare disorders there are many doctors caring for very few patients and is hard to define who the expert clinicians are. In DEB there is an added difficulty, as they require many different experts, from psychologists to surgeons. We defined experts as those clinicians caring for more than 10 patients with the disorder. To find them we localized doctors of main reference centers and asked them to identify other experts, we asked patients and patient association to identify missing experts. We also did a Medline search looking for authors of papers on DEB describing more than 3 patients in our country. Using this combined strategy we think that we have identified most experts in our country, and that our sample is highly representative of them, as all identified experts have participated in the PSP.

Finally, given the scarcity of participants it was difficult to schedule a meeting with participants balanced in terms of age, sex and severity that they could all attend. Two of the participants could not be at the meeting, and has to participate remotely, via teleconference (using Skype®). For researchers planning other PSPs, it can be interesting to notice that remote participation with the help of a moderator was feasible (only one remote assistant per group and both in the final global discussion), and that those participants felt integrated in the discussion process.

One of the weaknesses of this PSP might be the low number of participants (patients, HCPs and organizations) compared to other PSPs, which might jeopardize its generalizability. This would be true in absolute terms but it is important to take into account that prevalence of DEB is very low (7 cases per million) [33], the number of associations is scant and there are no many HCPs experts in treating DEB patients. Due to this rarity it was easy to approach most of the patients (because DEB is such a devastating disease, most of the patients and carers are members of DEBRA Spain) and experts in Spain. This, added to a high response rate (40%) when compared to other PSPs, makes the results very likely of being representative of the treatment uncertainties among patients and HCPs.

This PSP has been conducted in Spain, with some help from James Lind Alliance and following their methods. We do not think that the uncertainties found or the priorities are country specific, and thus they can be generalized to most settings. However, international aspects of the prioritization process advocated by the James Lind Alliance are worth further study, both in terms of improving methods to make international collaboration easier and to study whether results are reproducible and can be generalized to other countries.

## Conclusions

The final aim of this PSP was to uncover the uncertainties about DEB, and to prioritize them to fit the perceived needs of patients, carers and HCPs in this rare, but devastating, disease. We hope that the prioritized uncertainties are useful for researchers and funding bodies. PSP can be done in rare disorders, and are more important in these disorders as the budget for research is more limited.

## Abbreviations

AEDV: Spanish Academy of Dermatology and Venereology; DEB: Dystrophic Epidermolysis Bullosa; DEBRA: Dystrophic Epidermolysis Bullosa Research Association; HCPs: Health Care Professionals; JLA: James Lind Alliance; PICO: Patients Intervention, Comparison, Outcome; PSP: Priority Setting Partnerships; RCT: Randomized clinical trials; UK-DUETs: UK Database of Uncertainties about the Effects of Treatments

## Competing interest

The authors declared that they have no competing interests.

## Authors’ contributions

PDS participated in the design and coordination of the study, performed the statistical analysis and drafted the manuscript. AHM participated in the design and coordination of the study and helped to draft the manuscript. EMM participated in the design and coordination of the study. RL participated in the design and coordination of the study. ED participated in the design and coordination of the study. NR participated in the design and coordination of the study. MF participated in the design and coordination of the study. LC participated in the design and coordination of the study. BA participated in the coordination of the study. IGD conceived of the study and participated in its design and coordination, performed the statistical analysis and helped to draft the manuscript. All authors read and approved the final manuscript.
